# Crowding under scotopic and photopic vision in albino and normal-sighted participants

**DOI:** 10.1038/s41598-024-58369-0

**Published:** 2024-04-08

**Authors:** Avital Moshkovitz, Maria Lev, Uri Polat

**Affiliations:** School of Optometry and Vision Sciences, Faculty of Life Sciences, Bar-Ilan University, Ramat Gan, Israel

**Keywords:** Scotopic, Photopic, Crowding, Albino, Neuroscience, Sensory processing, Visual system

## Abstract

Crowding is a phenomenon in which the ability to recognize an object in a clutter deteriorates. It is, therefore, a fundamental aspect of object recognition and crucial in deciphering resolution. For visually impaired individuals, deficiency in crowding has a tremendous effect on vision and may reflect and predict the amount of deterioration in vision. It is well established that albinos suffer much more from crowding than normally sighted individuals under daylight luminance conditions. However, to our knowledge, this study is the first to investigate crowding in albino participants under low light conditions. In this study, we explored the crowding effect in a group of albino participants (n = 9) and a control group of normally sighted participants (n = 9). Crowding was conducted under daylight (photopic vision) and low light (scotopic vision). We measured the visual acuity threshold under crowding in three-letter spacing (0.5, 1, and 1.5) and compared it to a single target. Results indicate that albino participants experienced stronger crowding than the control under the photopic condition, while crowding under the scotopic condition was apparent in the albino but abolished for the control group. These findings highlight the importance of considering luminance when discussing the visually impaired population in general. In particular, it suggests that crowding in albinism is based on a peripheral-like mechanism and may indicate a cessation in visual development.

## Introduction

Crowding is a visual phenomenon that profoundly impacts vision^1^. It refers to the decline in object recognition ability when surrounded by visual clutter. In isolation, an object’s visibility relies on resolution limits. However, when surrounded by neighboring objects, their proximity diminishes the saliency of the central object, leading to reduced visibility^2^.

Crowding was considered mainly to occur primarily in the peripheral visual field of individuals with normal vision^3,4^. However, subsequent research has revealed that crowding can occur under specific conditions in the fovea—the central region of vision. These conditions include precise target locations measuring (4–6 arcs minutes)^2,5^, limited spacing between the target and surrounding masks (0.4 letters)^6,7^, and brief presentation times ranging from 30 to 240 ms^6,7^.

In contrast to individuals with normal vision, individuals with visual impairments, particularly those who experienced disruptions in the visual pathway during childhood (such as amblyopia), have been shown to experience crowding in both the peripheral and central visual fields^5,8–10^. Notably, individuals with albinism have consistently demonstrated robust crowding effects in the central visual field as well under daylight conditions of luminance^1,11–13^.

Albinism is a congenital condition characterized by heterogeneous disorders affecting melanin synthesis^14^. The prevalence of all forms of albino is 1 to 17,000^15^. Albinism mutation can be expressed as oculocutaneous albinism, which affects the entire body, or Ocular Albinism, which affects only the eyes^14^. The lack of pigmentation results in anatomical structure deficiencies such as foveal hypoplasia (underdeveloped fovea^16^), increased light sensitivity (photophobia), involuntary eye movements (nystagmus), and eyes misalignment (strabismus) in 53% of the cases^11^. These impairments contribute to reduced visual resolution, with visual acuity ranging between 6/18 and 6/120 and reduced contrast sensitivity^11,14,15,17–22^. In addition, studies have found that albinism has an amblyopia-like component affecting binocular vision and the symptoms accompanying their disease^22–24^.

Although there is extensive research on crowding under photopic (daylight) conditions for both normally sighted individuals and those with albinism, and some for mesopic conditions^25–29^, the literature regarding crowding under scotopic (night vision) conditions is limited. Night vision plays a crucial role in the visual system and is associated with reduced visual functions such as visual acuity, object recognition, and motion perception^30,31^. Numerous studies have investigated crowding during photopic conditions for normally sighted individuals^6,32^ as well as for populations with nystagmus^13,33,34^ and albinism^11,34^. However, fewer studies exploring crowding specifically under scotopic conditions were conducted, and those only for normally sighted individuals^1,11^.

Limited research has investigated the visual capabilities of individuals with strabismic amblyopia in conditions of reduced luminance (low light levels)^35,36^. These studies have revealed that under conditions of low luminance, the point at which visual information can no longer be distinguished (cutoff frequency) remains comparable to that of individuals without amblyopia (controls). This suggests that visual performance is contingent upon the complexity of the tasks being undertaken^35^. Moreover, in situations characterized by a substantial reduction in luminance, both the amblyopic eye and the non-amblyopic (fellow) eye experienced a reduction in visual acuity. Notably, the difference in visual acuity between the eyes was not statistically significant. Furthermore, this reduction in visual acuity was also found to be comparable to that observed in individuals with typical visual development^36^.

Our hypothesis revolves around the notion that individuals with albinism display elevated light sensitivity and photophobia and share resemblances in visual processing with amblyopia. Consequently, this similarity could contribute to divergent processing patterns in situations of low-light exposure. To the best of our knowledge, this study is the first to investigate the phenomenon of crowding in individuals with albinism under scotopic conditions. Our findings revealed that for the normally sighted individuals, moving from photopic to scotopic luminance resulted in crowding reduction. However, in the case of the albino participants, when moving from photopic to scotopic luminance crowding was maintained. These results have important implications for understanding the underlying mechanisms of central visual processing in individuals with albinism.

## Results

In this study, we investigated the perceptual processing of individuals with albinism using crowding experiments. We explored the impact of scotopic luminance compared to photopic luminance on their vision. The crowding experiment was performed under three letter-spacing (0.5, 1, and 1.5). Subsequently, the results of the albino group (n = 9) were compared to those of the normally sighted group (n = 9). Results are presented as the visual acuity threshold of the central target in degrees, determined using a staircase 3:1 design, which converged to a 79% correct response rate. The magnitude of the crowding effect was presented as threshold elevation and was calculated by subtracting the single-target result from the crowding condition. For multiple comparisons and interactions, see supplementary.

### Photopic condition

#### Normally sighted participants (control group)

**Figure 1:**
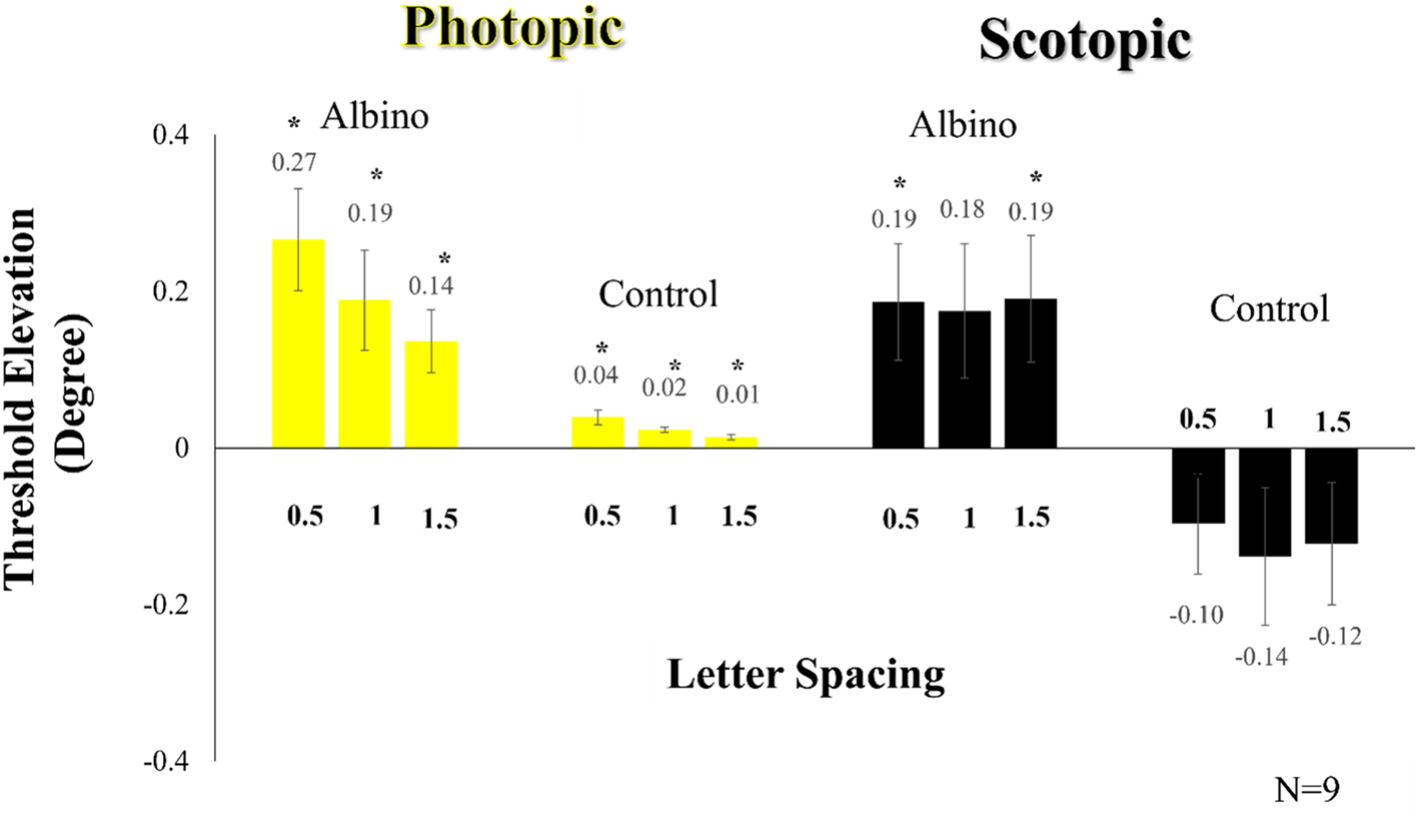
Crowding threshold elevation of the albino group vs. the control normally sighted participants under photopic (yellow bar) and scotopic (black bar) luminance. The crowding experiment was conducted in three-letter spacing conditions (0.5, 1, and 1.5). Error bars represent the standard error of the mean. Paired t-tests were conducted to present the statistical differences between the single target and the letter spacing conditions. *p ≤ 0.05 indicates statistical significance.

For the control participants under photopic luminance, typical crowding was observed. The results (mean ± SE) obtained for a single target, 0.5, 1, and 1.5 spacing were 0.077° (± 0.007°), 0.116° (± 0.01°), 0.1° (± 0.008°) and 0.09° (± 0.007°), respectively [VA between 6/5-to 6/8]. The differences between the single and crowded targets (letter spacing distances of 0.5, 1, and 1.5) were statistically significant. Threshold elevation was calculated for the control group under photopic luminance. Threshold elevation (mean ± SE) for the letter spacing distances (0.5, 1, and 1.5) were 0.039° (± 0.008°), 0.023° (± 0.003°), and 0.01° (± 0.003°), respectively, (p-values of p = 0.002, p < 0.001, and p = 0.004, respectively; paired *t* tests). Results are presented in Fig. 1.

#### Albino participants

For the albino participants under photopic luminance, we observed a significant increase in crowding, particularly notable for 0.5 letter spacing. The results (mean ± SE) obtained for a single target, 0.5, 1, and 1.5 spacing were 0.35° (± 0.04°), 0.62° (± 0.1°), 0.54° (± 0.09°), and 0.49° (± 0.07°), respectively [VA between 6/24-to 6/44]**.** The results revealed highly significant differences between the single target and the crowded conditions, i.e., the threshold elevation (means ± SE; were 0.266° (± 0.06°), 0.189° (± 0.06°), and 0.136° (± 0.038°) respectively. The differences between the single target and the crowded conditions were highly significant (p-values of 0.0025, 0.014, and 0.0072, respectively; paired t-tests).

The crowding level observed for 0.5 letter-spacing under photopic luminance significantly diverged from the control condition (F(1,16) = 13.374, p = 0.002; as determined by a one-way ANOVA, see sTable 1). These results may be derived from a stronger crowding effect in the albino participants. Threshold elevation results are presented in Fig. 1.

In the study, the exclusion criteria for the control group included individuals with normal visual development, with normal visual acuity, or acuity corrected to normal. This led to a narrower standard error in the control group compared with the albino group, where there is variability in visual acuity^14^, hence a larger standard error.

The results indicated a statistically significant difference between the single target and crowding, highlighting a distinct trend of crowding under scotopic luminance for albino participants. In contrast, the crowding results for the control group did not show statistically significant differences, yet the averages revealed a clear trend of reduced crowding in the control group.

### Scotopic condition

#### Normally sighted participants (control group)

For the control participants under scotopic luminance, the mean (± SE) results obtained for a single target, 0.5, 1, and 1.5 spacing were 1.01° (± 0.087°), 0.917° (± 0.074°), 0.876° (± 0.096°), 0.89° (± 0.1°), respectively [VA between 6/63 and 6/72]. Thus, the crowding in the control group was abolished under scotopic luminance. Furthermore, not only did the presence of a mask fail to interfere with the identification of the central target but there was a minor improvement in the identification of the central target across all crowded conditions compared to the single target. The differences between the single target and 0.5, 1, and 1.5 letter spacing under scotopic luminance were not statistically significantly different for all conditions. The mean (± SE) threshold elevation for 0.5, 1, and 1.5 letter spacing were − 0.097° (± 0.06°), − 0.139° (± 0.08°), and − 0.12° (± 0.07°), respectively. (p-values were p = 0.147, p = 0.133, and p = 0.134, respectively; paired *t* tests). Results are presented in Fig. 1.

#### Albino participants

For the albino participants under scotopic luminance, typical crowding was observed. The size (mean ± SE) of the single and crowded letter (spacing distances of 0.5, 1, and 1.5) were 1.14° (± 0.049°), 1.32° (± 0.114°), 1.31° (± 0.07°), and 1.32° (± 0.1°), respectively [VA between 6/82- and 6/95]. The threshold elevation (mean ± SE) for 0.5, 1, and 1.5 letter spacing were 0.186° (± 0.007°), 0.175° (± 0.08°), and 0.19° (± 0.08°), respectively. We found statistically significant differences between the results of the single target and the crowding condition of 0.5 and 1.5 letter spacing under scotopic luminance. (p-values for 0.5, 1, and 1.5 letter spacing were p = 0.029, p = 0.062, and p = 0.037, respectively; paired *t* test). Results are presented in Fig. 1.

To assess the differences in the crowding effect (threshold elevation) between the albino and control groups under scotopic luminance, a one-way ANOVA was performed. The analysis showed a significant effect with F(1, 16) = 9.342 and p = 0.008 (see sTable 1). The results suggest a stronger crowding effect in the albino participants compared to the control group.

##### Relationships between visual resolutions and crowding

The visual acuity of the albino population was assessed with an ETDRS chart in logMar units under photopic luminance. The average VA was 0.73 (± 0.22 STD). Pearson correlation coefficients were computed to assess the relationship between visual acuity for the albino and the experimental conditions (single target, 0.5, 1, and 1.5 letter spacing).

**Figure 2:**
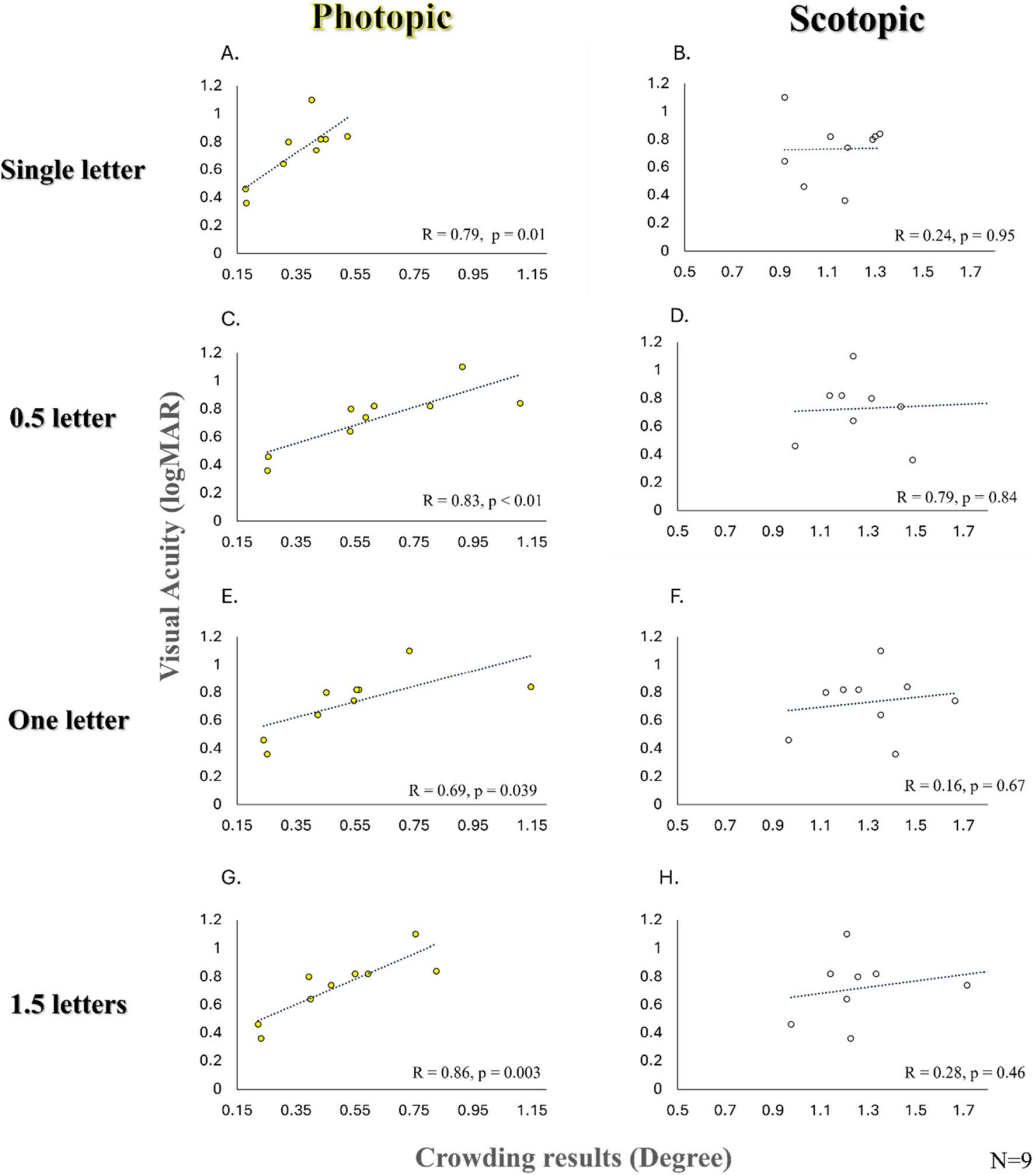
Scatter plots illustration depicting the relationship between photopic ETDRS chart visual acuity (Y-axis) and the experiment results in degrees (X-axis) within the albino group across various crowding conditions (single target, 0.5, 1, and 1.5 letter spacing) under both photopic (Left) and scotopic (Right) luminance. The Pearson correlation coefficient is reported to assess the strength of the correlation, along with the corresponding statistical results (p-value). Notably, a stronger correlation was observed under photopic luminance conditions. In the current study, the exclusion criteria for the control group included individuals with normal visual development and either normal visual acuity or acuity corrected to normal. This led to narrower standard deviations in the control group compared with the albino group, where there is variability in visual acuity^14^.

Under photopic luminance, the visual acuity results showed a strong positive correlation with all experimental conditions: single target (R = 0.79, p = 0.01), 0.5 letter-spacing (R = 0.83, p = 0.006), 1 letter-spacing (R = 0.69, p = 0.039), and 1.5 letter-spacing (R = 0.86, p = 0.003). These findings illustrate a statistically significant strong correlation between the actual visual acuity (logMAR) and the experimental visual threshold results under crowding conditions in the albino group under photopic luminance (see Fig. 2). This result is consistent with Bonnhe, Sagi & Polat, regarding the correlation between VA and crowding^8^.

Furthermore, Pearson correlation coefficients were calculated to assess the relationship between visual acuity and crowding (single target, 0.5, 1, and 1.5 letters) under scotopic luminance. The correlation coefficients obtained were as follows: single target (R = 0.24, p = 0.95), 0.5 letter spacing (R = 0.79, p = 0.84), 1 letter spacing (R = 0.16, p = 0.67), and 1.5 letter spacing (R = 0.28, p = 0.46). Among these conditions, the strongest correlation was observed at 0.5 letter spacing. However, none of the correlations reached statistical significance, suggesting different underlying mechanisms may influence visual acuity under scotopic luminance conditions. Surprisingly, when examining the decrease in the resolution under the low luminance, the group that mainly suffered from resolution reduction in consequences of luminance reduction was the control, not the albino group (See Fig. 2).

For the control group, the average VA was − 0.068 (± 0.088 STD). Pearson correlation coefficients were calculated to assess the relationship between visual acuity and crowding (single target, 0.5, 1, and 1.5 letters) under photopic luminance. The correlation coefficients obtained were as follows: single target (R = 0.5, p = 0.17), 0.5 letter spacing (R = 0.36, p = 0.34), 1 letter spacing (R = 0.36, p = 0.34), and 1.5 letter spacing (R = 0.28, p = 0.47) under scotopic luminance the correlation coefficients obtained were as follows: single target (R = 0.36, p = 0.34), 0.5 letter spacing (R = 0.15, p = 0.69), 1 letter spacing (R = -0.18, p = 0.65), and 1.5 letter spacing (R = 0.16, p = 0.69). There seem to be some correlations between visual acuity and crowding under specific conditions, but these correlations are generally weak and often not statistically significant. While the variance (STD) in the VA of the albino group was spread and equal to two lines in the ETDRS chart, The variance of the result for the control was narrowed and limited to less than one line. This may suggest why the relationship between these variables may not be strong for the control group.

Among albino individuals, considerable variability in resolution exists across participants under both photopic and scotopic conditions. Notably, under scotopic conditions, the decrease in resolution affects each patient in a distinct manner that may not be predicted from the photopic condition; hence, it reflects a widespread and weak correlation. This observation underscores our hypothesis that a distinct mechanism underlies the processing of scotopic luminance rather than a simple reduction in luminance achieved through filtering.

## Discussion

On a daily basis, we encounter low luminance experiences when engaging in outdoor activities at nighttime or indoor activities in dark environments. Night vision plays a fundamental role in visual processing.

This study explored the mechanism underlying visual processing under low-light (scotopic) stimuli for albino participants compared to daylight (photopic) stimuli. We examined the visual processing utilizing a crowding experiment, which is an essential factor influencing functional vision and everyday life^1^. We compare crowding at the center of the visual field between albino and normal-vision participants. Results for the albino participants under photopic luminance exhibited a potent crowding effect under all conditions. The crowding effect was more substantial than the control under the same conditions. Compared to the normal control group, the powerful crowding effect under photopic luminance is well established in former literature^1,11^ and can be explained by excessive eye movement due to nystagmus^12,33^, strabismus^11^, and amblyopia-like components^22–24,37^. However, when investigating crowding under scotopic luminance, in the albino group, the crowding was also intense, while the control group showed no crowding effect.

Crowding has long been explained by different theories, from low to high hierarchy of visual processing and even physical explanation^2^. One of the first theories is the ‘physiological model’^38–40^. According to this model, the visual cortex receives information from different parts of the retina and integrates the information within the receptive field. The psychophysical visual units, the perceptive fields (PF), corresponded to the receptive field in the visual cortex^38,39,41^. This model suggests crowding occurs when a low-level PF integrates overlapping objects, such as the target and the flankers. Former studies showed that the crowding effect is correlated with the RF’s size, showing increased crowding with increased PF^42–44^.

The control results revealed typical crowding in the central visual field across all conditions under photopic luminance. Notably, the crowding effect was particularly pronounced when the letter spacing was set at 0.5, and it gradually decreased as the letter spacing distance increased. These findings are consistent with numerous studies that have demonstrated crowding in the central visual field during short durations and small letter distances (4–6 arcmin) under photopic stimuli^2,6,7,10^. Previous studies exploring the impact of letter-spacing on crowding in individuals with normal vision found that the most pronounced effect occurs with half-letter-spacing, and that crowding diminishes as the letter-spacing increases to one letter^6,32^. Thus, crowding decreases with increasing letter-spacing^32^. This phenomenon may be linked to the size of the perceptive field^3,6,45^, as evidenced in studies involving visually impaired individuals with amblyopia and albinism, where a significant crowding effect was observed not only at 0.5 letter-spacing but also at one letter-spacing (under photopic conditions). This effect is suggested to result from a larger perceptive field and arrested visual development^3,6,8,11,45^. Under photopic luminance conditions for both groups, crowding was most pronounced at 0.5 letter-spacing, decreasing in 1 and 1.5 letter-spacings. In controls, strong crowding was not found at 1 and 1.5 letter-spacing, beyond the suppression range^6,32^, and indeed, the effect was negligible (0.04–0.02 degree).

In contrast to the albino findings under scotopic luminance, our study revealed the absence of crowding effect for all letter-spacing conditions in the control group. Moreover, not only was the crowding abolished, but we found that the detection of the central letter was facilitated.

Under scotopic conditions, in both groups, larger receptive fields may be activated, leading to increased lateral interactions between neighboring neurons; hence, the effect of crowding is expected to increase. However, we found a lack of crowding for the control group. This aligns with former studies demonstrating the correlation between decreased luminance and diminished crowding and lateral interaction (a single letter surrounded by flanks)^26,28,46^.

In a separate research study conducted in our laboratory Moshkovitz^47^, we performed crowding experiments on individuals with typical visual development under scotopic luminance. We employed a distinct experimental methodology that measured sensitivity^47^. Despite utilizing differing methodologies to gauge crowding effects, our results remained remarkably congruent with those observed under photopic and scotopic luminance. Under conditions of photopic illumination, crowding was present, and it was again abolished for scotopic luminance.

Subsequently, the study mentioned above^47^ thoroughly investigated similar conditions that could clarify the absence of crowding in low-light scenarios, specifically low-contrast crowding measurements. In the study, crowding under low contrast was measured. The study's results indicated that low-contrast stimuli exhibited a reduced crowding effect at the participant's contrast threshold compared with slightly higher contrast stimuli^47^. This reduction in the crowding effect due to low contrast at the threshold was also observed in previous research, thus corroborating our results^12,48,49^. Hence, it is possible that under scotopic conditions, the target and the surroundings were perceived as low contrast- near the threshold, thus reducing the effect of crowding. In this regard, it could be possible that the albino group adapted to low lighting and had a higher sensitivity to darkness. Another possible explanation for the absence of crowding in the control group is related to spatial interactions in the human retina, which are not restricted solely to photopic stimuli but also occur during scotopic luminance^50^. Under low-light conditions, the receptive fields of ganglion cells in the dark-adapted retina display slightly broader central fields with weaker responses in the surrounding areas^51^.

One possible explanation for the absence of crowding in the control group is related to spatial interactions in the human retina, which are not restricted solely to photopic stimuli but also occur during scotopic luminance^50^. Under low-light conditions, the receptive fields of ganglion cells in the dark-adapted retina display slightly broader central fields with weaker responses in the surrounding areas^51^.

We hypothesized that processing stimuli in the central visual field under low luminance, reaching a high threshold level of visual perception resembles how the central visual field processes non-optimal stimuli under low-luminance, which may result in weak neuronal activation within the excitation range, potentially explaining the observed effects of crowding diminished.

We suggest that the increase of PF can provide an explanation for the intense crowding effect observed in albino participants, which may result from non-normal development during the critical period^37^. A recent study has shown that the PF’s size in children is larger than in adults up to age 6, the age of maturation of visual processing^44^, hence, children have more crowding. Study (Bonneh, Sagi & Polat)^52^ shows a large crowding effect in the fovea of strabismic amblyopia, a condition that is modeled as the periphery of normal vision^52^. Thus, we suggest that individuals with abnormal and underdeveloped visual systems might experience arrested development, leading to increased PF’s size and, consequently, a more pronounced crowding effect under both low and high luminance conditions.

We posit that the crowding phenomenon observed in our results aligns primarily with the physiological model. Flom et al.^38,40,53^ proposed a limitation regarding the extent of crowding, suggesting a correlation with the size of the receptive field (RF). Specifically, in the periphery, where the RF is large, crowding tends to increase^53^. It is assumed that the visual functions of the fovea in children resemble those of the adult’s periphery and mature over time. The size of a perceptive field is matured at the age of 5–6 years old, thus influencing crowding during childhood development^54,55^. In studies on albinism, it is apparent that visual development is arrested, resembling human infants in several visual aspects, such as low visual acuity and an underdeveloped fovea^37^. Our findings suggest that crowding has a lasting impact, causing suboptimal performance not only during childhood but also persisting well beyond the maturation of single-letter acuity^54,56^. Thus, our results for an increased crowding effect in albino individuals are consistent with an increased foveal perceptive field resembling extra foveal processing in the control group. Contrary to controls, our results indicate that albino individuals exhibit functions resembling peripheral mechanisms under both high- and low-light conditions, in line with the physiological theory. we propose that the mechanism responsible for processing low-luminance stimuli in these individuals may behave differently compared to those with normally developed visual systems.

Contemporary crowding models support a two-stage processing approach. The initial stage involves simple feature detection, potentially occurring in V1, whereas the second stage is crucial for feature integration or object interpretation beyond V1. Despite indications of top-down effects in crowding, the exact role of attention in this process remains unclear^2^.

Scotopic luminance reduced resolution for all groups. However, the effect across letter-spacings was insufficient to detect differences. It is assumed ^37^ that in albino individuals, the mechanism underlying foveal processing is arrested, resembles that of the children, and the generation of robust lateral inhibition, resulting in strong crowding at larger letter-spacing. In contrast, the mechanism underlying foveal processing for controls has reduced lateral inhibition for large letter-spacing, resulting in less crowding.

In addition, it is important to highlight the difference in sensitivity between the two groups in this context. First, the subjects were all healthy with normal or corrected to normal visual acuity. Hence, a narrower variety (a small standard error) compared with the albino group was found. Second, when comparing the decrease in visual acuity from photopic to scotopic luminance, the control group experienced a greater reduction in visual acuity, indicating that they were likely tested closer to their visual acuity limit. Conversely, the albino participants might require a stronger reduction in luminance to reach the limit of their sensitivity hold, and it is possible that their visual processing under stronger scotopic conditions could resemble that of the control group.

## Conclusion

To our best knowledge, this is the first study that explored crowding in albino population under scotopic luminance. The fact that the crowding was present only in the albino group under low luminance may imply distinct neurological mechanisms in processing information in the center of the visual field. This difference manifests as increased difficulty in navigation under low-light conditions, primarily attributed to stronger crowding effects^57^. Standard visual function examinations that conducted in clinical settings under photopic luminance can pose serious risks, particularly when considering activities like driving in low-light conditions. Thus, comprehensive visual examinations under low-light is especially crucial for individuals with professions that require nighttime activities, such as police officers, firefighters, and soldiers.

## Methods

### Participants

The study included a total of 18 adult participants, consisting of nine individuals with normal or corrected-to-normal vision (mean age: 26.44 ± 8.26 STD) and nine with albinism (one with Ocular albinism and eight with Oculocutaneous albinism; mean age: 32.1 ± 6.77 STD). The age-matching between the nystagmus and normally sighted groups was not statistically significantly different (*t* test, p = 0.24).

The study protocol received approval from the Internal Review Board (IRB) of Bar-Ilan University, and informed consent was obtained from all subjects. All methods employed in the study followed the applicable guidelines and regulations.

### Experimental set-up

The psychophysical experiments in this study utilized a custom platform developed by Yoram S. Bonneh^58^. Stimuli were displayed on an Eizo FG-2421, 24″ HD monitor operating at a refresh rate of 120 Hz. The screen size was 52 by 30 cm (18.43° × 11.31°) with a resolution of 1920 × 1080 pixels. Gamma correction was calibrated to a luminance of 30cd/m^2^.

### Stimuli

The stimuli used in the experiment consisted of a single-centered black letter ‘E’ and 5 × 5 matrices of ‘E’ letters displayed on a gray background. The inter-letter spacing distances were set to 0.5, 1, and 1.5 times the width of a single letter. The crowding exposure was set to 120 ms.

During the experiment, participants were required to report the direction (e.g. left or right) of the centered ‘E’ letter. A central fixation point was provided to facilitate gaze fixation on the target. The size of the ‘E’ letters used in the experiment was determined using a staircase 3:1 design, which converged to a 79% correct response rate^59^. This method increased the target size by 0.1 log units (26%) after an incorrect response or when the smallest target size was reached. The smallest target size was programmed to appear as 10 pixels on the screen to prevent any distortion in the appearance of the ‘E’ letter.

In young individuals with normal vision, foveal crowding occurs within a brief duration, typically ranging from 40 to 120 milliseconds^6,43,60^. Nevertheless, studies on albino individuals and those who are visually impaired have revealed that it challenges the visual temporal processing as well, requiring an extended exposure time to reach normal processing^8,22^. Because a presentation time longer than 120 ms in the control group may eliminate crowding^6^, the optimal presentation time for testing the crowding effect in each group was set at 120 ms.

To minimize fatigue or any discomfort, participants were allowed to pause and rest as much as needed after each response.

Each experimental condition was repeated up to a maximum of 40 trials to establish the threshold. The maximum number of trials indicates the upper limit of trials to reach threshold within the staircase procedure. For albino participants, the average was 34 trials, and for the control it was 36, indicating that the threshold was reached before reaching the maximum upper limit.

The experiment was performed three times, resulting in an average of up to 40X3 repetitions for each condition. The experiment was carried out binocularly, considering the input from both eyes.

**Figure 3:**
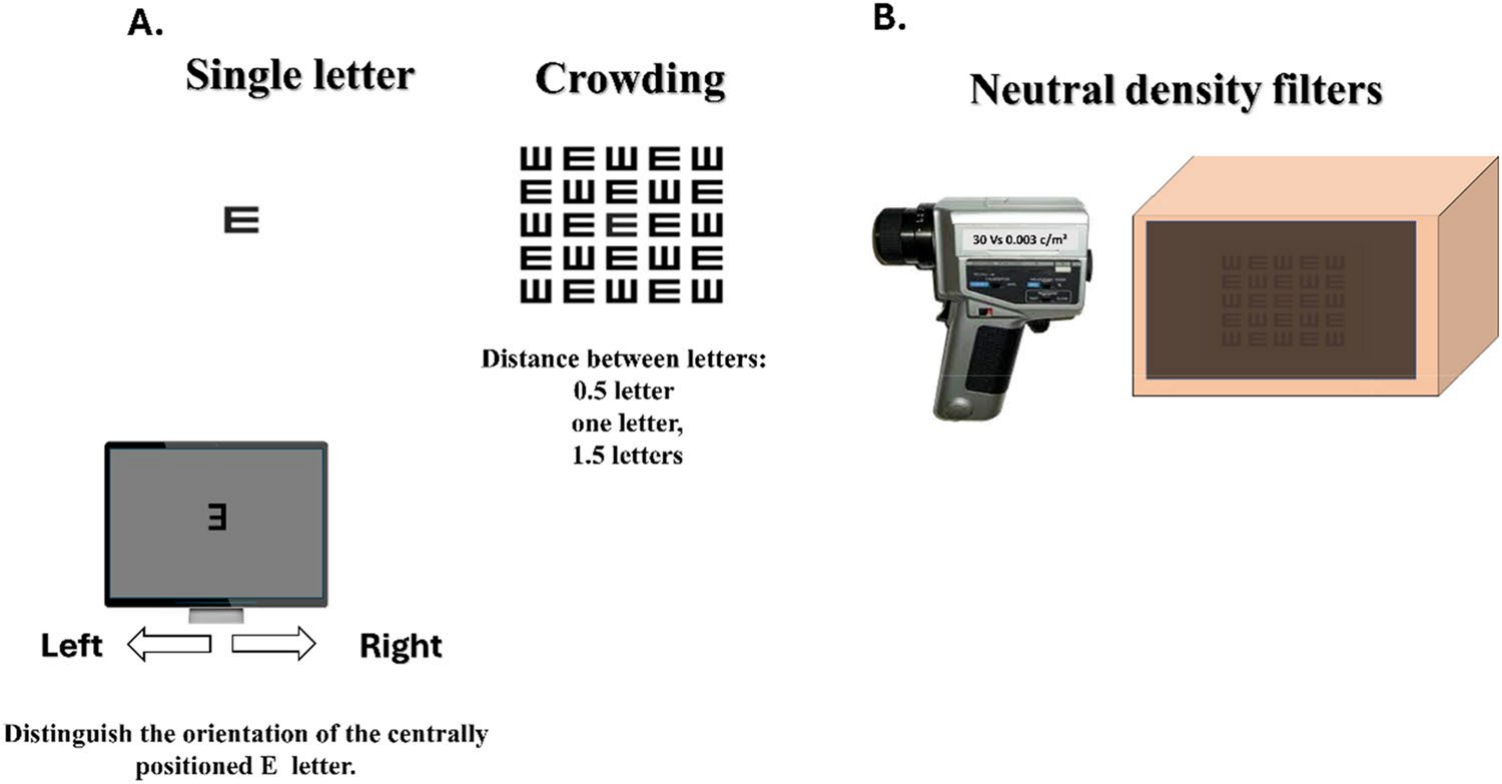
The crowding experiments. An E-letter target is presented as a single presentation or a matrix with 0.5, 1, and 1.5. The task was to discriminate E letter orientation (right and left directions). A photopic light-level experiment was conducted under a luminance of 30 c/m^2^. Low-light level experiments were conducted using neutral density filters (Rosco Laboratories) and measured using a luminance meter.

This study was performed equally under photopic (daylight) and scotopic (night vision) luminance levels. Photopic luminance was fixed to 30 cd/m^2^, which corresponds to the photopic definition above 3.4 cd/m^2^^61^. Scotopic luminance was fixed at 0.003, which is below the threshold of 0.034 cd/m^2^^61^. We used neutral density filters (Rosco Laboratories, Stamford, USA) to control the luminance levels. We measured the luminance using an LS-100 luminance meter (KONICA MINOLTA) (see Fig. 3). The study took place in a specially designed and supervised isolated room adapted to a dark environment and luminance was monitored. This level of luminance simulates daily life for typical individuals, however, the experience may be different for those individuals with albinism due to their impaired visual functions. These differences are investigated in this study.

Prior to the scotopic task, participants underwent a 15-min dark adaptation period. The maximal adaptation of cone cells to darkness occurs between 2 and 10 min, whereas rod cells' adaptation is longer and undergoes changes over time. After 7 min, there is a rapid improvement in rod sensitivity, followed by a gradual decline after 15 min^62^. The response of individuals with albinism to low to moderate flash luminance remained within the anticipated normal range^63^. Therefore, all participants underwent a 15-min dark adaptation period that covers the foveal adaptation time of all individuals, along with additional time to enable extra foveal adaptation.

For the albino group, the sitting distance from the screen was 1.5 m for both photopic and scotopic luminance conditions. In the control group, the sitting distance during the scotopic experiment was also 1.5 m from the screen. However, for the photopic experiment, the sitting distance was adjusted to 2.66 m due to the resolution limitations of the screen and stimuli. The guiding principle of resolution is consistency across all viewing distances, ensuring a uniform visual angle regardless of the viewing distance. Regarding an individual with normal vision (20/20 visual acuity) positioned 6 m from the computer screen, the letters occupy a visual angle of 5 min of arc^64,65^, corresponding to 0.873 cm. However, when the distance is reduced to 3 m, if the letter size is maintained, it results in a larger visual angle that is not equivalent to the 5 arcminutes visual angle. To address this problem, there is a need to adjust the size of the 20/20 line and reduce it to 0.581 cm. In our study, to prevent distortion of the letter ‘E’ on the screen (to avoid not enough pixels per letter), we programmed the smallest target to be displayed using 10 pixels, reflecting a visual angle of 0.1 degrees from 1.5 m. Since this letter size did not align with the need for 5 arcminutes of visual angle for 20/20 vision, we subsequently changed the distance to 2.66 m, and obtained a new visual angle of 0.058 degrees. Thus, to ensure the correct resolution, we modified the sitting distance from the screen.

### Data analysis

The results were initially obtained in pixels and then converted to visual degrees. The magnitude of the crowding effect was presented as threshold elevation and was calculated by subtracting the single-target result from the crowding condition.

We utilized a one-way ANOVA test with the group variable (control/albino) as the independent measure and the delta values represented the difference between the single target and crowding at 0.5 letters under both photopic and scotopic conditions as the dependent variables (sTables 1, 2). The main effects were tested for each table separately for multiple comparisons using FDR^66^; sTable 1.

Paired Student’s *t* tests were utilized to evaluate the statistical differences between tasks.

The correlation between visual acuity measured using the ETDRS chart and the threshold size in degrees across all experimental conditions and light levels was evaluated using the Pearson correlation coefficient with a 95% confidence interval.

### Supplementary Information

Supplementary Information.

## Data Availability

The datasets used and/or analyzed during the current study are available from the corresponding author on reasonable request.
