# Supplementary material for: Crowding under scotopic and photopic vision in albino and normal-sighted participants
Source: Sci Rep. 2024 Apr 8;14:8234. doi: 10.1038/s41598-024-58369-0 (PMC11001935; doi:10.1038/s41598-024-58369-0)
Supplement: Supplementary file 1 — Supplementary Information. [file 41598_2024_58369_MOESM1_ESM.docx]

Crowding under scotopic and photopic vision in albino and normal-sighted participants

Avital Moshkovitz, Maria Lev and Uri Polat

School of Optometry and Vision Sciences, Faculty of Life Sciences, Bar-Ilan University

# Supplementary

We provide further elaboration on the statistical analysis in the supplementary sections. One-way analysis of variance (ANOVA) was conducted to examine the effects of the independent variables, 'group' (Albino/control), on the dependent variables: single and crowding at 0.5 letter spacing and the delta between them for photopic and scotopic luminance sTable 1. The main effects were tested for each table separately for multiple comparisons using FDR (Benjamini & Hochberg, 1995); all p-values remained statistically significant.

|  | **One -Way ANOVA** | | | | | | | |
| --- | --- | --- | --- | --- | --- | --- | --- | --- |
|  | | | Sum of Squares | df | Mean Square | F | Sig. | FDR Correction Sig. |
| Single letter under scotopic luminance | | Between Groups | .066 | 1 | .066 | 1.519 | .236 | .236 |
|  |  | Within Groups | .690 | 16 | .043 |  |  |  |
|  |  | Total | .756 | 17 |  |  |  |  |
| Crowding: Half letter under scotopic luminance | | Between Groups | .734 | 1 | .734 | 13.780 | .002 | .008 |
|  |  | Within Groups | .852 | 16 | .053 |  |  |  |
|  |  | Total | 1.585 | 17 |  |  |  |  |
| Single letter under photopic luminance | | Between Groups | .353 | 1 | .353 | 48.116 | .000 | .000 |
|  |  | Within Groups | .117 | 16 | .007 |  |  |  |
|  |  | Total | .470 | 17 |  |  |  |  |
| Crowding: Half letter under photopic luminance | | Between Groups | 1.156 | 1 | 1.156 | 28.388 | .000 | .000 |
|  |  | Within Groups | .651 | 16 | .041 |  |  |  |
|  |  | Total | 1.807 | 17 |  |  |  |  |
| Delta (half letter minus one letter under photopic luminance | | Between Groups | .232 | 1 | .232 | 13.374 | .002 | .008 |
|  |  | Within Groups | .277 | 16 | .017 |  |  |  |
|  |  | Total | .509 | 17 |  |  |  |  |
| Delta (half letter minus one letter under  Scotopic luminance | | Between Groups | .361 | 1 | .361 | 9.342 | .008 | .016 |
|  |  | Within Groups | .618 | 16 | .039 |  |  |  |
|  |  | Total | .978 | 17 |  |  |  |  |

sTable 1: One-way analysis of variance (ANOVA) was conducted to examine the effects of the independent variables, 'Group' (Albino/control), on the dependent variables: single and crowding at 0.5 letter spacing and the delta between them for photopic and scotopic luminance. The ANOVA table results show the F-statistic, degrees of freedom (df), and p-values for significance for each factor and the interaction effect. The main effects were tested for each table separately for multiple comparisons using FDR (Benjamini & Hochberg, 1995); all p-values remained statistically significant.

Analysis of Variance (three way ANOVA) was conducted to examine the effects of the independent variables included ‘Group’ (Albino/control), luminance conditions (photopic/scotopic), and letter distance conditions (0.5, 1, and 1.5), whereas the dependent variable was the overall result. The variables ‘Group’ and ‘Luminance’ were found to be significant <0.001. A significant interaction, group * conditions (p=0.012), was identified. The main effects were tested for each table separately for multiple comparisons using FDR (Benjamini & Hochberg, 1995); the p-values remained statistically significant only for Group and Luminance. See sTable 2. Multiple Comparisons Post Hoc Test conducted to determine which specific groups differ from each other. See sTable3

|  | **Tests of the Between-Subjects Effects** | | | | | | |
| --- | --- | --- | --- | --- | --- | --- | --- |
|  |  | | | | | | |
| Source | | Type III Sum of Squares | df | Mean Square | F | Sig. | FDR Correction Sig |
| group | | 3.601 | 1 | 3.601 | 89.953 | .000 | .000 |
| luminance | | 16.951 | 1 | 16.951 | 423.476 | .000 | .000 |
| conditions | | .179 | 2 | .089 | 2.230 | .113 | .565 |
| group * luminance | | .056 | 1 | .056 | 1.400 | .240 | .720 |
| group * conditions | | .368 | 2 | .184 | 4.598 | .012 | .565 |
| luminance * conditions | | .059 | 2 | .030 | .741 | .479 | .958 |
| group * luminance * conditions | | .025 | 2 | .013 | .314 | .731 | .958 |
| Error | | 3.843 | 96 | .040 |  |  |  |
| Total | | 77.948 | 108 |  |  |  |  |
| Corrected Total | | 25.081 | 107 |  |  |  |  |
|  | a. R Squared = .847 (Adjusted R Squared = .829) | | | | | | |

sTable 2: Analysis of Variance (Three way ANOVA) was conducted. The independent variables included groups (Control/Albino), luminance conditions (photopic/scotopic), and letter distance conditions (0.5, 1, and 1.5), whereas the dependent variable was the overall result. The variables Group and Luminance were found to be significant <0.001. A significant interaction, group * conditions (p=0.012), was identified. The main effects were tested for each table separately for multiple comparisons using FDR (Benjamini & Hochberg, 1995); the p-values remained statistically significant only for Group and Luminance.

| **Multiple Comparisons Post Hoc Test** | | | | | | | |
| --- | --- | --- | --- | --- | --- | --- | --- |
| Dependent Variable: results | | | | | | | |
|  | (I) conditions | (J) conditions | Mean Difference (I-J) | Std. Error | Sig. | 95% Confidence Interval | |
|  |  |  |  |  |  | Lower Bound | Upper Bound |
| Tukey HSD | single | half | -.0985 | .04716 | .098 | -.2108 | .0138 |
|  |  | one | -.0620 | .04716 | .390 | -.1743 | .0502 |
|  | half | single | .0985 | .04716 | .098 | -.0138 | .2108 |
|  |  | one | .0365 | .04716 | .720 | -.0758 | .1487 |
|  | one | single | .0620 | .04716 | .390 | -.0502 | .1743 |
|  |  | half | -.0365 | .04716 | .720 | -.1487 | .0758 |
| LSD | single | half | -.0985^*^ | .04716 | .039 | -.1921 | -.0049 |
|  |  | one | -.0620 | .04716 | .192 | -.1556 | .0316 |
|  | half | single | .0985^*^ | .04716 | .039 | .0049 | .1921 |
|  |  | one | .0365 | .04716 | .441 | -.0571 | .1301 |
|  | one | single | .0620 | .04716 | .192 | -.0316 | .1556 |
|  |  | half | -.0365 | .04716 | .441 | -.1301 | .0571 |
| Bonferr-oni | single | half | -.0985 | .04716 | .118 | -.2134 | .0164 |
|  |  | one | -.0620 | .04716 | .575 | -.1769 | .0529 |
|  | half | single | .0985 | .04716 | .118 | -.0164 | .2134 |
|  |  | one | .0365 | .04716 | 1.000 | -.0784 | .1514 |
|  | one | single | .0620 | .04716 | .575 | -.0529 | .1769 |
|  |  | half | -.0365 | .04716 | 1.000 | -.1514 | .0784 |
| Based on the observed means.  The error term is Mean Square(Error) = .040. | | | | | | | |
| *. The mean difference is significant at the 0.05 level. | | | | | | | |

sTable 3: Post hoc analyses were conducted for the interaction effect, group * conditions (p=0.012). A significant effect was identified through the LSD test when comparing single and half conditions.
